# The Accuracy of Prostate Cancer Localization Diagnosed on Transrectal Ultrasound-Guided Biopsy Compared to 3-Dimensional Transperineal Approach

**DOI:** 10.1155/2013/249080

**Published:** 2013-12-29

**Authors:** Kevin Krughoff, Khadijah Eid, Jason Phillips, Diliana Stoimenova, Daniel Smith, Colin O'Donnell, E. David Crawford, Al Barqawi

**Affiliations:** Department of Surgery, Division of Urology, University of Colorado Denver School of Medicine, Academic Office One Building, Mail Stop C-319, 12631 East 17th Avenue, Room L15-5602, Aurora, CO 80045, USA

## Abstract

*Background*. Prostate cancer is often understaged following 12-core transrectal ultrasound- (TRUS-) guided biopsies. Our goal is to understand where cancers are typically missed by this method. *Methods*. Transperineal 3-dimensional mapping biopsy (3DMB) provides a more accurate depiction of disease status than transrectal ultrasound- (TRUS-) guided biopsy. We compared 3DMB findings in men with prior TRUS-guided biopsies to determine grade and location of missed cancer. Results were evaluated for 161 men with low-risk organ confined prostate cancer. *Results*. The number of cancer-positive biopsy zones per patient with TRUS was 1.38 ± 1.21 compared to 3.33 ± 4.06 with 3DMB, with most newly discovered cancers originating from the middle lobe and apex. Approximately half of all newly discovered cancerous zones resulted from anterior 3DMB sampling. Gleason upgrade was recognized in 56 patients using 3DMB. When both biopsy methods found positive cores in a given zone, Gleason upgrades occurred most frequently in the middle left and right zones. TRUS cancer-positive zones not confirmed by 3DMB were most often the basal zones. *Conclusion*. Most cancer upgrades and cancers missed from TRUS biopsy originated in the middle left zone of the prostate, specifically in anterior regions. Anterior sampling may lead to more accurate diagnosis and appropriate followup.

## 1. Introduction 

An increasing proportion of prostate cancer is being diagnosed at clinically insignificant stages, raising controversy surrounding overdiagnosis and overtreatment of a growing number of patients with potentially nonmortal yet psychologically morbid disease [1]. Data seem to suggest that Watchful Waiting (WW) or Active Surveillance protocols (AS) should be the method of choice in many or even most low-risk situations due to the long natural history of prostate cancer and the generally favorable prognosis for clinically localized noninvasive disease [2–4]. There now exists substantial evidence that an observational approach would be a practical, cost saving, and quality-of-life preserving option for many [5].

The proportion of men with clinically localized prostate cancer (LPC) who could potentially benefit from AS has risen from approximately 25% in the early 1990s to almost 50% as of 2007 [6]; however the proportion of men on AS has remained largely unchanged for over a decade. Only 6.8% of men with prostate cancer opt for AS according to the CaPSURE database. This level has fluctuated somewhat over the years but has changed only slightly since 2000 when it was at 6.2% [6, 7]. The vast majority of low risk patients pursue surgical, radiological, and hormonal interventions, and this carries substantial implications on patient quality of life and the healthcare system as a whole given the significant risks of urinary and sexual side effects that come with these treatment modalities [8].

Poor AS enrollment may be attributable to limitations in our ability to evaluate the true extent of cancer at the individual level and predict progression [9]. Large and Eggener concluded after studying data pertaining to AS that more accuracy in identifying cancer grade and stage upon diagnosis would help AS gain more widespread acceptance [10]. Supporting Large and Eggener's conclusion is an analysis of radical prostatectomy specimens from men who were previously on AS, finding that most cancer progression was a result of undersampling during the initial biopsy [11]. In a more recent review of the advances in patient selection methods and treatment triggers for AS, Lees et al. also reported that more sophisticated biopsy schemes could have serious implications for advancing patient selection in AS [12].

One such biopsy scheme described by Lees et al. is a comprehensive 3-dimensional mapping biopsy (3DMB) procedure developed by Barqawi et al. that demonstrates substantial improvements in accuracy over the standard transrectal ultrasound guided (TRUS) biopsy. 3DMB is a comprehensive biopsy of the whole prostate gland. The prostate is placed within a virtual 3D grid and all biopsies are taken systematically at 5 mm interval in *x*/*y*/*z*-registered coordinates. Overall the risk of missing any cancer in any of the prostate regions is significantly lower than that reported for TRUS biopsy which is around 25–30% [13]. Their initial report on the use of 3DMB found that a significant portion of 180 men diagnosed with low-risk disease harbored clinically significant cancers, resulting in approximately a quarter of them receiving an upgraded cancer diagnosis and half receiving an upstaged diagnosis [13].

Since its inception, researchers have demonstrated the ability of 3DMB to more accurately detect and grade prostate cancer [1]. It remains to be seen, however, where cancers are being missed in standard office TRUS-guided procedures compared to 3DMB findings, and whether these missed cancers lead to diagnostic upgrades necessitating more aggressive treatment. An understanding of where prostate cancer is being missed by traditional 12-core TRUS-guided biopsy procedures carries implications for not only reducing understaging, but also reducing overtreatment if a more accurate and reliable diagnostic methodology is pursued. Thus, the aim of the research presented here is to evaluate the differences in cancer location obtained by 3DMB and TRUS biopsy methods in an effort to highlight potential trends in the location of missed cancers and cancer foci that result in upstaged and upgraded cancer.

## 2. Methods

Patients are seen at our institution for mapping biopsies due to recurrent negative biopsies in the presence of rising PSA, preference for watchful waiting but more risk assessment data, and/or pursuit of focal therapy (in which case 3DMB is standard of care). Our study is a Colorado Institutional Review Board (COMIRB) approved retrospective comparison of our 3DMB results with prior TRUS biopsy results for men aged 40–85 years with either low-risk organ confined prostate cancer or high suspicion for cancer who were seen at our institution between 2006 and 2009. Low-risk classification was based on regionally defined TRUS report demonstrating Gleason 3 + 4 = 7 or lower, ≤50% tumor burden, and a PSA <10 ng/dL. 161 men met the inclusion criteria; of those, 68% (*n* = 110) had mapping biopsies performed by urologist AB and the remaining 32% (*n* = 51) had mapping biopsies performed by urologist EDC. Approximately half of the TRUS biopsies were performed in house and half were performed at tertiary care centers. Most patients had TRUS and 3DMB performed within the same year; however, the maximum duration allowed between biopsies was 3 years.

As 3DMB coordinates are not readily translated into the zones typically used in TRUS reports, we divided 3DMB coordinates into six locations for comparison: Left Apex (LA), Left Mid (LM), Left Base (LB), Right Apex (RA), Right Mid (RM), and Right Base (RB). These are homologous with the locations reported in standard 12-core TRUS biopsies. Collected data included age, prostate size, number of days between biopsy procedures, use of 5-alpha-reductase inhibitors (5-ARIs), number of cores taken, and zones of the prostate positive for cancer.

Transperineal mapping biopsy was performed based on the 5 mm grid method using triplane transrectal ultrasound with accessory probe stabilization under general anesthesia with patients in the high dorsal lithotomy position. The ultrasound has the capability to have stepwise linear analogous or equivalent to the brachytherapy model. Two gold fiducial markers we placed at the time of 3DMB serve as an orientation landmark for future potential diagnostic or treatment considerations. All specimens from the biopsy of the prostate were marked according to their topographical location within the prostate and sent individually for pathological reporting. Total operating time is on average 60 minutes, whereas 3DMB biopsy time itself ranges from 15 to 25 minutes. Statistical analysis was performed in SAS 9.2.

## 3. Results

Of the 161 men enrolled in this study, the mean age was 61.6 ± 7.73, and the mean duration between TRUS and 3DMB for each patient was 192.18 ± 287.96 days, the mean prostate size was 49.34 ± 20.65 grams at time of TRUS biopsy and 39.25 ± 15.25 grams at the time of 3DMB. Prostate size at time of 3DMB ranged from 17 to 95 grams. Fifty-three subjects were taking 5-ARIs during the time between TRUS-guided biopsy and 3DMB; the mean prostatic volume reduction among these patients was 8.01 ± 15.2 grams.

Mean number of cores taken for 3DMB was 61.4 ± 22.69, ranging between 27 and 124. The mean number of positive cores per mapping biopsy was 5.25 ± 4.81. The median positive number of zones per patient using TRUS-guided biopsy was 1 (IQR = 0.63 to 2.13) compared to a median number of positive zones for 3DMB of 2 (IQR = 0.59 to 6.10). The amount of positive zones found from 3DMB was greater and statistically significant than the amount of positive zones found from TRUS-guided biopsy (3.33 versus 1.38, *P* < 0.0001).

Following TRUS-guided biopsy, 75% of patients (*n* = 121) were found to have Gleason 6 cancer and 7.5% (*n* = 12) were found to have Gleason 7 cancer. Following 3DMB, 54% of patients (*n* = 87) were found to have Gleason 6 cancer, 25% (*n* = 40) were found to have Gleason 7 cancer, 4% (*n* = 7) were found to have Gleason 8 cancer, and 2% (*n* = 3) were found to have Gleason 9 cancer (Table 1).

Twenty-eight patients (17.4%) were found to be cancer-free following TRUS-guided biopsy compared to 24 (14.9%) following 3DMB. Of the 24 patients who were reported cancer-free by 3DMB, 11 had a prior positive TRUS-guided biopsy. Of the 28 patients who reported cancer-free by initial TRUS-guided biopsy, 18 were found to be cancer-positive on subsequent 3DMB. Of the cancers missed by 3DMB, 12.9% were Gleason 3 + 4 = 7, and 87.1% were Gleason 3 + 3 = 6. Of the cancers missed by TRUS, 60.3% were Gleason 3 + 3 = 6, 24.9% were Gleason 3 + 4 = 7, 4.4% were Gleason 4 + 4 = 8, and 2% was Gleason 4 + 5 = 9.

There were 68 instances in which the LM zone was recorded cancer-positive by 3DMB in the face of prior negative TRUS-guided biopsy results, followed by 62 instances in the RM zone and 41 instances in the LA zone. Thus, most newly discovered cancers originated from the middle of the left lobe, middle of the right lobe, and apex of left lobe. Approximately one-half of all newly discovered cancerous zones were diagnosed as a result of anterior 3DMB core sampling. In our sample, 56 patients experienced an overall Gleason upgrade (34.8%), with newly discovered Gleason 7 findings following a similar distribution pattern as newly discovered Gleason 6 findings. For cases where both biopsy methods found positive cores in a given zone, Gleason upgrades occurred most frequently in the middle left lobe. The TRUS cancer-positive zones that were most often unconfirmed for cancer by 3DMB were the base of the right lobe and the base of the left lobe. In cases where unilateral cancer was found on TRUS biopsy, 3DMB reported cancer on the contralateral side of the prostate for approximately 28.5% (*n* = 46) of patients (Tables 2 and 3).

Of the 161 men enrolled, one patient experienced an episode of atrial fibrillation during anesthesia. One patient experienced contact dermatitis probably due to the antiseptic used during the mapping biopsy. One patient with a history of DVTs presented to the clinic 8 days after his mapping biopsy with a DVT. One patient complained of incontinence the day after his mapping procedure, and this was resolved with oxybutynin. Complication rates for these procedures have been described previously [13].

## 4. Discussion

It has been demonstrated previously that 3DMB provides a more accurate depiction of prostate tumor burden and the presence of contralateral tumors due to more extensive sampling, methodical biopsy spacing, and access to the anterior region of the prostate [13, 14]. Our data demonstrates that the majority of prostate cancers missed by TRUS but detected by 3DMB as well as the majority of cancer stage upgrades appear to originate in the middle of the left and right zones of the prostate, with a significant proportion of newly discovered cancers derived from anterior core sampling.

The data suggest poor agreement between 12-core TRUS biopsy and 3DMB results with regard to staging and grading of prostate cancer, most notably in the aforementioned regions of the prostate. While our data demonstrate a high relative positive predictive and negative predictive value for cancer presence versus no presence when comparing TRUS biopsy to 3DMB, the lack of concordance regarding location of cancer, findings of contralateral cancer, and discovery of greater amounts and higher grades of cancer raise concern. The relatively high rates of cancer upgrading upon 3DMB are in accordance with previous studies [13]. These results also support conclusions reached from whole mount radical prostatectomy specimen studies and repeat biopsy studies which demonstrated higher levels of anterior region cancers amongst patients with previous negative TRUS biopsies [15]. Research performed by Duffield et al. on RP specimens from patients for whom AS failed also suggests that increased sampling in the anterior region is necessary [16].

The importance of anterior core sampling follows, given the difficulties associated with accessing anterior regions of the prostate using the transrectal approach, along with the fact that approximately 1/5 of carcinomas are found to lie anterior to the urethra in RP specimens [17]. MRI imaging modalities have progressed through the years in relation to prostate cancer detection and evaluation and this may also assist in anterior zone sampling [18, 19]. Current studies seem to demonstrate its potential; however, the data is still preliminary [20].

While other studies evaluating cancer location have employed the use of sectioned specimens, we did not feel that this was appropriate in our circumstances due to the volume loss and change in core position upon prostatectomy. Limitations of this study surround the consistency of TRUS biopsy, given the fact that approximately half of the TRUS biopsy records included in this study were in house, whereas the other half came from tertiary centers. Another concern regards the use of 5-alpha-reductase-inhibitors (5-ARIs), which were employed to shrink the volume of the prostate when the pubic arch obstructed transperinneal access. Some research suggests that 5-ARIs, by shrinking the prostate, actually highlight the presence of high grade disease, thus heightening the sensitivity of diagnostic tests like 3DMB [21]. The specific effect of 5-ARIs on the diagnostic location and grade of prostate cancer as determined by 3DMB has yet to be determined. Systemic improvements in prostate cancer detection and AS enrollment must come with sensible systemic changes, and a large component of this will be cost. Thus, a cost-effectiveness analysis of prostate cancer diagnostic techniques will serve greatly in the effort to avoid overdiagnosis and overtreatment. It should be noted that repeat of TRUS biopsy may yield additional cancer at the same rate of 3DMB, however localization is more difficult to determine and advancements in anterior sampling may still be necessary.

As of this study, more than 700 mapping biopsies have been performed at our institution without any documented urinary retention referring to the time period following the first 48 hours after the procedure. All patients have a catheter for 24–48 hours following the procedure, the duration of which is determined by the operator who takes into account preprocedure lower urinary tract symptoms. Pre-3DMB urinary tract symptoms related to BPH returned to baseline within a week. Patients who undergo 3DMB are advised to take tamsulosin hydrochloride 10 days after the procedure or to continue existing BPH medications. In our experience, the number of mapping biopsy cores did not affect postoperative recovery; however, patients with preexisting lower urinary tract symptoms had longer recovery times.

## 5. Conclusion

Overtreatment is an increasingly important issue in the field of prostate biopsy and more accurate cancer detection has the potential to increase the utility of AS and reduce the amount of overtreatment, while at the same time providing appropriate treatment for those who may go understaged due to insufficient sampling. The majority of prostate cancers missed by TRUS but detected by 3DMB as well as most cancer upgrades appear to originate in the middle of the left lateral and right lateral zones of the prostate, specifically in anterior regions. Attention should be given to this region of the prostate when evaluating for prostate cancer in order to improve biopsy accuracy and avoid undersampling. The potential for missed cancer in this region is significant, which suggests that efforts made to address anterior prostate sampling may lead to improved outcomes and enrollment in AS.

## Figures and Tables

**Table 1:** Gleason grade distribution.

Gleason	TRUS	3DMB
0	28	24
6	121	87
7	12	40
8	0	7
9	0	3

**Table 2:** 3DMB and TRUS-guided biopsy characteristics.

	TRUS	3DMB
Total cores taken per patient	12	
Number of positive cores per patient	1.38 (±1.21)	
	Patients	Patients
LA positive	55/161 (34.2%)	71/161 (44.1%)
LM positive	48/161 (29.8%)	109/161 (67.7%)
LB positive	40/161 (24.8%)	37/161 (23.0%)
RA positive	43/161 (26.7%)	54/161 (33.5%)
RM positive	52/161 (32.3%)	104/161 (64.4%)
RB positive	39/161 (24.2%)	32/161 (19.9%)

**Table 3:** Newly discovered cancers and cancer upgrades.

	LA	LM	LB	RA	RM	RB
Instances of cancer positive cores coming from TRUS-determined cancer negative zones (total instances = 244)	41 (16.8%)	68 (27.9%)	19 (7.8%)	35 (14.3%)	62 (25.4%)	19 (7.8%)
Proportion of newly cancerous zones resulting from anterior 3DMB cores (total instances = 108)	17 (41.5%)	28 (41.2%)	8 (42.1%)	17 (48.6%)	25 (40.3%)	13 (68.4%)
Instances of positive Gleason 7 cores (total instances = 178)	32 (18%)	43 (24.7%)	13 (7.3%)	24 (13.5%)	56 (31.5%)	10 (5.6%)
Gleason upgrade instances by zone (3 + 4 = 7, 4 + 3 = 7, or higher) by zone(s) (*N* = 56, total instances = 98)	18 (18.4%)	28 (28.6%)	4 (4.1%)	14 (14.3%)	28 (28.6%)	6 (6%)
Instances of positive Gleason 7 cores for zones which both biopsy methods reported as cancer positive	3	8	1	2	4	0
Instances of unconfirmed cancer by zone upon 3DMB (total instances = 99)	17 (17.2%)	8 (8%)	20 (20.2%)	18 (18.2%)	12 (12.1%)	24 (24.3%)
